# (*E*)-Methyl 3-(3,5-dibromo-2-hy­droxy­benzyl­idene)carbazate

**DOI:** 10.1107/S1600536810033799

**Published:** 2010-08-28

**Authors:** Lu-Ping Lv, Songhui Liu

**Affiliations:** a Linjiang College, Hangzhou Vocational and Technical College, Hangzhou 310018, People’s Republic of China

## Abstract

The title compound, C_9_H_8_Br_2_N_2_O_3_, crystallizes with two very similar independent mol­ecules in the asymmetric unit, each of which adopts a *trans* configuration with respect to the C=N bond. Intra­molecular O—H⋯N hydrogen bonds are observed in each independent mol­ecule. In the crystal structure, mol­ecules are linked into chains propagating along [010] by N—H⋯O and C—H⋯O hydrogen bonds. In addition, C—H⋯π inter­actions stabilize the structure.

## Related literature

For general background to benzaldehyde­hydrazone derivatives, see: Parashar *et al.* (1988 ▶); Hadjoudis *et al.* (1987 ▶); Borg *et al.* (1999 ▶); Kahwa *et al.* (1986 ▶); Santos *et al.* (2001 ▶). For a related structure, see: Shang *et al.* (2007 ▶).
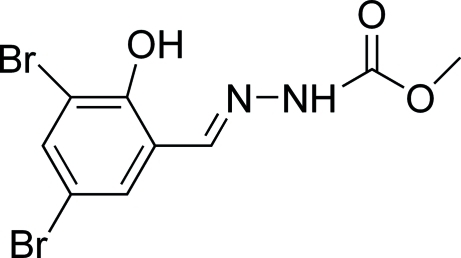


## Experimental

### 

#### Crystal data

C_9_H_8_Br_2_N_2_O_3_
*M* *_r_* = 351.99
Triclinic, 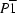

*a* = 7.6907 (11) Å
*b* = 9.9886 (14) Å
*c* = 15.503 (2) Å
α = 92.254 (6)°
β = 95.647 (6)°
γ = 91.394 (6)°
*V* = 1183.8 (3) Å^3^
*Z* = 4
Mo *K*α radiation
μ = 6.84 mm^−1^
*T* = 223 K
0.22 × 0.21 × 0.18 mm

#### Data collection

Bruker SMART CCD area-detector diffractometer
Absorption correction: multi-scan (*SADABS*; Bruker, 2002 ▶) *T*_min_ = 0.977, *T*_max_ = 0.989
12305 measured reflections
4109 independent reflections
2425 reflections with *I* > 2σ(*I*)
*R*_int_ = 0.059

#### Refinement

*R*[*F* ^2^ > 2σ(*F* ^2^)] = 0.055
*wR*(*F* ^2^) = 0.175
*S* = 1.01
4109 reflections
289 parameters
H-atom parameters constrained
Δρ_max_ = 0.63 e Å^−3^
Δρ_min_ = −0.62 e Å^−3^

### 

Data collection: *SMART* (Bruker, 2002 ▶); cell refinement: *SAINT* (Bruker, 2002 ▶); data reduction: *SAINT*; program(s) used to solve structure: *SHELXS97* (Sheldrick, 2008 ▶); program(s) used to refine structure: *SHELXL97* (Sheldrick, 2008 ▶); molecular graphics: *SHELXTL* (Sheldrick, 2008 ▶); software used to prepare material for publication: *SHELXTL*.

## Supplementary Material

Crystal structure: contains datablocks I, global. DOI: 10.1107/S1600536810033799/ci5169sup1.cif

Structure factors: contains datablocks I. DOI: 10.1107/S1600536810033799/ci5169Isup2.hkl

Additional supplementary materials:  crystallographic information; 3D view; checkCIF report

## Figures and Tables

**Table 1:** Hydrogen-bond geometry (Å, °) *Cg*1 and *Cg*2 are centroids of the C1–C6 and C10–C15 rings, respectively.

*D*—H⋯*A*	*D*—H	H⋯*A*	*D*⋯*A*	*D*—H⋯*A*
O1—H1⋯N1	0.82	1.88	2.604 (7)	146
N2—H2⋯O6	0.86	2.01	2.845 (6)	163
N4—H4⋯O2^i^	0.86	2.00	2.828 (6)	161
O4—H4*A*⋯N3	0.82	1.89	2.609 (7)	145
C7—H7⋯O6	0.93	2.56	3.312 (8)	139
C16—H16⋯O2^i^	0.93	2.52	3.282 (8)	139
C9—H9*B*⋯*Cg*1^ii^	0.97	2.89	3.594 (8)	131
C18—H18*C*⋯*Cg*2^iii^	0.97	2.89	3.561 (8)	128

Symmetry codes: (i) 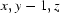; (ii) 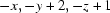; (iii) 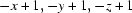.

**Fig. 1.**
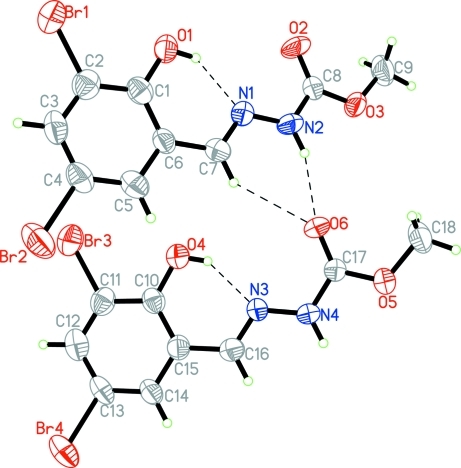
The asymmetric unit of the title compound. Displacement ellipsoids are drawn at the 50% probability level. Dashed lines indicate hydrogen bonds.

**Fig. 2.**
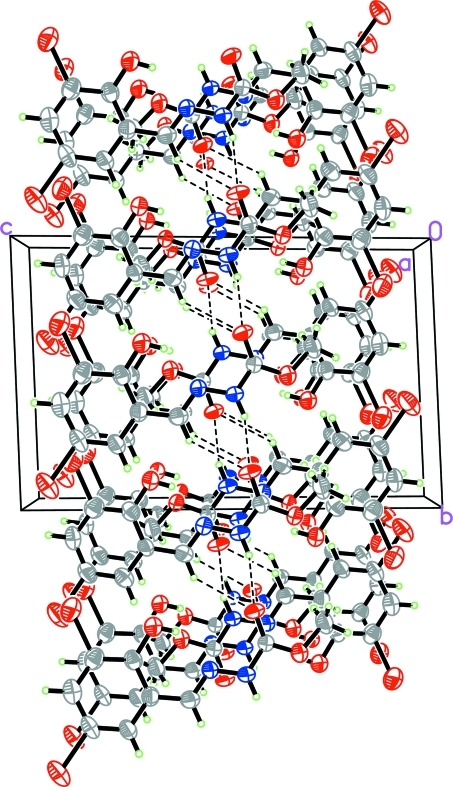
Crystal packing of the title compound. Hydrogen bonds are shown as dashed lines.

## supplementary crystallographic information

### Comment

Benzaldehydehydrazone derivatives have attracted much attention due to their pharmacological activity (Parashar *et al.*, 1988) and their photochromic properties (Hadjoudis *et al.*, 1987). They are important intermediates of 1,3,4-oxadiazoles, which have been reported to be versatile compounds with many interesting properties (Borg *et al.*, 1999). Metal complexes based on Schiff bases have received considerable attention because they can be utilized as model compounds of active centres in various proteins and enzymes (Kahwa *et al.*, 1986; Santos *et al.*, 2001). We report here the crystal structure of the title compound (Fig. 1).

The title compound contains two independent, but almost identical molecules in the asymmetric unit. Each independent molecule adopts a *trans* configuration with respect to the C═N bond. The N1/N2/O2/O3/C7-C9 and N3/N4/O5/O6/C16-C18 planes form dihedral angles of 8.18 (6)° and 7.28 (7)°, respectively, with the C1—C6 and C10—C15 planes. The bond lengths and angles are comparable to those observed for methyl*N*'-[(*E*)-4-methoxybenzylidene]hydrazinecarboxylate (Shang *et al.*, 2007). Intramolecular O—H···N hydrogen bonds are observed in each independent molecule.

In the crystal structure, the molecules are linked into chains running along the [010] by N—H···O and C—H···O hydrogen bonds. In addition, intermolecular C—H···π interactions are observed, which further stabilize the structure (Table 1).

### Experimental

3,5-Dibromo-2-hydroxybenzaldehyde (2.79 g, 0.01 mol) and methyl hydrazinecarboxylate (0.90 g, 0.01 mol) were dissolved in stirred methanol (15 ml) and left for 5.5 h at room temperature. The resulting solid was filtered off and recrystallized from ethanol to give the title compound in 96% yield. Single crystals suitable for X-ray analysis were obtained by slow evaporation of an ispropanol solution at room temperature (m.p. 415–417 K).

### Refinement

H atoms were positioned geometrically (O–H = 0.83 Å, N-H = 0.87 Å and C–H = 0.94 or 0.97 Å) and refined using a riding model, with *U*_iso_(H) = 1.2*U*_eq_(C,N) and 1.5*U*_eq_(C_methyl_).

### Crystal data

**Table:**

C_9_H_8_Br_2_N_2_O_3_	*Z* = 4
*M**_r_* = 351.99	*F*(000) = 680
Triclinic, *P*1	*D*_x_ = 1.975 Mg m^−^^3^
Hall symbol: -P 1	Mo *K*α radiation, λ = 0.71073 Å
*a* = 7.6907 (11) Å	Cell parameters from 4109 reflections
*b* = 9.9886 (14) Å	θ = 1.3–25.0°
*c* = 15.503 (2) Å	µ = 6.84 mm^−^^1^
α = 92.254 (6)°	*T* = 223 K
β = 95.647 (6)°	Block, colourless
γ = 91.394 (6)°	0.22 × 0.21 × 0.18 mm
*V* = 1183.8 (3) Å^3^	

### Data collection

**Table:**

Bruker SMART CCD area-detector diffractometer	4109 independent reflections
Radiation source: fine-focus sealed tube	2425 reflections with *I* > 2σ(*I*)
graphite	*R*_int_ = 0.059
φ and ω scans	θ_max_ = 25.0°, θ_min_ = 1.3°
Absorption correction: multi-scan (*SADABS*; Bruker, 2002)	*h* = −9→8
*T*_min_ = 0.977, *T*_max_ = 0.989	*k* = −11→11
12305 measured reflections	*l* = −18→18

### Refinement

**Table:**

Refinement on *F*^2^	Primary atom site location: structure-invariant direct methods
Least-squares matrix: full	Secondary atom site location: difference Fourier map
*R*[*F*^2^ > 2σ(*F*^2^)] = 0.055	Hydrogen site location: inferred from neighbouring sites
*wR*(*F*^2^) = 0.175	H-atom parameters constrained
*S* = 1.01	*w* = 1/[σ^2^(*F*_o_^2^) + (0.0947*P*)^2^] where *P* = (*F*_o_^2^ + 2*F*_c_^2^)/3
4109 reflections	(Δ/σ)_max_ = 0.001
289 parameters	Δρ_max_ = 0.63 e Å^−^^3^
0 restraints	Δρ_min_ = −0.62 e Å^−^^3^

### Special details

**Table:**

Geometry. All esds (except the esd in the dihedral angle between two l.s. planes) are estimated using the full covariance matrix. The cell esds are taken into account individually in the estimation of esds in distances, angles and torsion angles; correlations between esds in cell parameters are only used when they are defined by crystal symmetry. An approximate (isotropic) treatment of cell esds is used for estimating esds involving l.s. planes.
Refinement. Refinement of F^2^ against ALL reflections. The weighted R-factor wR and goodness of fit S are based on F^2^, conventional R-factors R are based on F, with F set to zero for negative F^2^. The threshold expression of F^2^ > 2sigma(F^2^) is used only for calculating R-factors(gt) etc. and is not relevant to the choice of reflections for refinement. R-factors based on F^2^ are statistically about twice as large as those based on F, and R- factors based on ALL data will be even larger.

### Fractional atomic coordinates and isotropic or equivalent isotropic displacement parameters (Å^2^)

**Table:**

	*x*	*y*	*z*	*U*_iso_*/*U*_eq_	
C1	0.0209 (8)	0.9946 (6)	0.2567 (4)	0.0463 (16)	
C2	−0.0373 (9)	1.0058 (7)	0.1701 (5)	0.0551 (18)	
C3	−0.0724 (9)	0.8941 (7)	0.1156 (5)	0.0578 (19)	
H3	−0.1108	0.9037	0.0568	0.069*	
C4	−0.0513 (9)	0.7688 (7)	0.1474 (5)	0.0568 (19)	
C5	0.0050 (8)	0.7525 (7)	0.2340 (5)	0.0529 (18)	
H5	0.0167	0.6661	0.2554	0.063*	
C6	0.0441 (8)	0.8656 (6)	0.2893 (4)	0.0428 (15)	
C7	0.1087 (8)	0.8455 (6)	0.3787 (4)	0.0485 (16)	
H7	0.1129	0.7586	0.3999	0.058*	
C8	0.2901 (8)	1.0311 (6)	0.5634 (4)	0.0454 (16)	
C9	0.4215 (10)	1.0965 (7)	0.7025 (5)	0.064 (2)	
H9A	0.4648	1.0566	0.7562	0.096*	
H9B	0.3295	1.1578	0.7135	0.096*	
H9C	0.5162	1.1451	0.6795	0.096*	
C10	0.3833 (8)	0.4911 (6)	0.2572 (4)	0.0436 (15)	
C11	0.4111 (8)	0.4998 (7)	0.1704 (5)	0.0522 (17)	
C12	0.4301 (8)	0.3882 (7)	0.1186 (5)	0.0582 (19)	
H12	0.4482	0.3955	0.0599	0.070*	
C13	0.4218 (9)	0.2622 (7)	0.1556 (5)	0.0528 (18)	
C14	0.3968 (8)	0.2485 (6)	0.2395 (4)	0.0495 (17)	
H14	0.3928	0.1627	0.2623	0.059*	
C15	0.3769 (8)	0.3617 (6)	0.2927 (4)	0.0436 (15)	
C16	0.3449 (8)	0.3454 (6)	0.3820 (4)	0.0465 (16)	
H16	0.3494	0.2596	0.4048	0.056*	
C17	0.2301 (8)	0.5304 (6)	0.5620 (4)	0.0427 (15)	
C18	0.1545 (10)	0.5951 (7)	0.7015 (5)	0.063 (2)	
H18A	0.1356	0.5552	0.7559	0.094*	
H18B	0.0492	0.6388	0.6790	0.094*	
H18C	0.2503	0.6607	0.7110	0.094*	
N1	0.1604 (7)	0.9473 (5)	0.4288 (4)	0.0483 (14)	
N2	0.2264 (7)	0.9247 (5)	0.5120 (4)	0.0542 (15)	
H2	0.2274	0.8442	0.5313	0.065*	
N3	0.3108 (7)	0.4455 (5)	0.4302 (3)	0.0452 (13)	
N4	0.2761 (7)	0.4238 (5)	0.5131 (4)	0.0508 (14)	
H4	0.2832	0.3443	0.5340	0.061*	
O1	0.0559 (6)	1.1076 (4)	0.3070 (3)	0.0547 (12)	
H1	0.0934	1.0875	0.3567	0.082*	
O2	0.2888 (7)	1.1459 (4)	0.5407 (3)	0.0688 (15)	
O3	0.3523 (6)	0.9915 (4)	0.6398 (3)	0.0557 (12)	
O4	0.3642 (6)	0.6050 (4)	0.3056 (3)	0.0547 (12)	
H4A	0.3491	0.5857	0.3559	0.082*	
O5	0.1974 (6)	0.4908 (4)	0.6391 (3)	0.0569 (12)	
O6	0.2219 (7)	0.6444 (4)	0.5383 (3)	0.0630 (14)	
Br1	−0.06212 (11)	1.17836 (8)	0.12394 (5)	0.0693 (3)	
Br2	−0.09610 (12)	0.61188 (8)	0.07391 (6)	0.0806 (3)	
Br3	0.41876 (12)	0.67238 (8)	0.12264 (6)	0.0735 (3)	
Br4	0.43692 (12)	0.10563 (8)	0.08150 (6)	0.0764 (3)	

### Atomic displacement parameters (Å^2^)

**Table:**

	*U*^11^	*U*^22^	*U*^33^	*U*^12^	*U*^13^	*U*^23^
C1	0.043 (4)	0.051 (4)	0.046 (5)	−0.004 (3)	0.010 (3)	−0.005 (3)
C2	0.053 (4)	0.060 (4)	0.054 (5)	0.003 (3)	0.009 (4)	0.007 (4)
C3	0.062 (4)	0.076 (5)	0.035 (4)	0.004 (4)	0.010 (3)	−0.001 (4)
C4	0.063 (4)	0.057 (4)	0.047 (5)	−0.007 (3)	0.000 (4)	−0.014 (4)
C5	0.053 (4)	0.053 (4)	0.052 (5)	0.007 (3)	0.009 (3)	−0.009 (3)
C6	0.052 (4)	0.042 (4)	0.035 (4)	0.000 (3)	0.013 (3)	−0.003 (3)
C7	0.061 (4)	0.039 (3)	0.047 (5)	0.004 (3)	0.011 (3)	−0.002 (3)
C8	0.061 (4)	0.042 (4)	0.034 (4)	0.003 (3)	0.012 (3)	0.000 (3)
C9	0.076 (5)	0.076 (5)	0.038 (5)	0.003 (4)	0.000 (4)	−0.008 (4)
C10	0.040 (3)	0.051 (4)	0.040 (4)	0.005 (3)	0.007 (3)	−0.002 (3)
C11	0.050 (4)	0.061 (4)	0.049 (5)	0.003 (3)	0.013 (3)	0.011 (4)
C12	0.053 (4)	0.078 (5)	0.043 (5)	0.001 (4)	0.006 (3)	−0.002 (4)
C13	0.054 (4)	0.063 (5)	0.040 (5)	0.004 (3)	0.008 (3)	−0.018 (3)
C14	0.061 (4)	0.049 (4)	0.039 (4)	−0.001 (3)	0.010 (3)	−0.008 (3)
C15	0.046 (4)	0.047 (4)	0.038 (4)	0.003 (3)	0.005 (3)	0.002 (3)
C16	0.053 (4)	0.039 (3)	0.048 (5)	0.001 (3)	0.008 (3)	0.003 (3)
C17	0.058 (4)	0.036 (4)	0.034 (4)	0.000 (3)	0.004 (3)	0.002 (3)
C18	0.079 (5)	0.070 (5)	0.040 (5)	0.000 (4)	0.015 (4)	−0.009 (4)
N1	0.067 (4)	0.042 (3)	0.036 (4)	0.002 (3)	0.002 (3)	0.003 (3)
N2	0.083 (4)	0.028 (3)	0.050 (4)	0.002 (3)	0.001 (3)	0.006 (3)
N3	0.066 (4)	0.037 (3)	0.034 (3)	0.003 (2)	0.013 (3)	0.001 (2)
N4	0.083 (4)	0.029 (3)	0.041 (4)	0.003 (3)	0.010 (3)	0.003 (3)
O1	0.079 (3)	0.048 (3)	0.037 (3)	−0.005 (2)	0.004 (2)	0.004 (2)
O2	0.114 (4)	0.025 (2)	0.067 (4)	−0.003 (2)	0.004 (3)	0.013 (2)
O3	0.091 (3)	0.041 (2)	0.032 (3)	−0.003 (2)	−0.005 (2)	0.005 (2)
O4	0.078 (3)	0.047 (3)	0.041 (3)	0.008 (2)	0.014 (2)	0.004 (2)
O5	0.087 (3)	0.049 (3)	0.037 (3)	0.008 (2)	0.016 (2)	0.002 (2)
O6	0.110 (4)	0.030 (2)	0.053 (3)	0.010 (2)	0.021 (3)	0.011 (2)
Br1	0.0837 (6)	0.0719 (5)	0.0532 (6)	0.0081 (4)	0.0047 (4)	0.0159 (4)
Br2	0.0933 (7)	0.0793 (6)	0.0647 (6)	0.0018 (5)	−0.0013 (5)	−0.0291 (5)
Br3	0.0975 (7)	0.0730 (6)	0.0543 (6)	0.0022 (4)	0.0234 (5)	0.0158 (4)
Br4	0.0873 (6)	0.0814 (6)	0.0595 (6)	0.0082 (4)	0.0131 (4)	−0.0301 (4)

### Geometric parameters (Å, °)

**Table:**

C1—O1	1.353 (7)	C11—C12	1.367 (9)
C1—C2	1.383 (9)	C11—Br3	1.905 (7)
C1—C6	1.412 (8)	C12—C13	1.406 (10)
C2—C3	1.378 (9)	C12—H12	0.94
C2—Br1	1.899 (7)	C13—C14	1.346 (9)
C3—C4	1.370 (9)	C13—Br4	1.917 (6)
C3—H3	0.94	C14—C15	1.393 (8)
C4—C5	1.387 (10)	C14—H14	0.94
C4—Br2	1.906 (7)	C15—C16	1.445 (9)
C5—C6	1.400 (8)	C16—N3	1.272 (7)
C5—H5	0.94	C16—H16	0.94
C6—C7	1.448 (9)	C17—O6	1.211 (7)
C7—N1	1.286 (7)	C17—O5	1.319 (7)
C7—H7	0.94	C17—N4	1.357 (7)
C8—O2	1.212 (7)	C18—O5	1.460 (7)
C8—O3	1.312 (8)	C18—H18A	0.97
C8—N2	1.355 (8)	C18—H18B	0.97
C9—O3	1.456 (7)	C18—H18C	0.97
C9—H9A	0.97	N1—N2	1.368 (7)
C9—H9B	0.97	N2—H2	0.87
C9—H9C	0.97	N3—N4	1.364 (7)
C10—O4	1.357 (7)	N4—H4	0.87
C10—C11	1.388 (9)	O1—H1	0.83
C10—C15	1.426 (8)	O4—H4A	0.83
			
O1—C1—C2	118.9 (6)	C11—C12—C13	118.1 (7)
O1—C1—C6	122.2 (6)	C11—C12—H12	120.9
C2—C1—C6	118.9 (6)	C13—C12—H12	120.9
C3—C2—C1	121.3 (7)	C14—C13—C12	122.3 (6)
C3—C2—Br1	119.1 (6)	C14—C13—Br4	119.6 (6)
C1—C2—Br1	119.6 (5)	C12—C13—Br4	118.0 (5)
C4—C3—C2	119.8 (7)	C13—C14—C15	119.8 (6)
C4—C3—H3	120.1	C13—C14—H14	120.1
C2—C3—H3	120.1	C15—C14—H14	120.1
C3—C4—C5	121.0 (6)	C14—C15—C10	119.4 (6)
C3—C4—Br2	121.1 (6)	C14—C15—C16	119.3 (6)
C5—C4—Br2	117.9 (5)	C10—C15—C16	121.3 (6)
C4—C5—C6	119.5 (6)	N3—C16—C15	120.9 (6)
C4—C5—H5	120.3	N3—C16—H16	119.5
C6—C5—H5	120.3	C15—C16—H16	119.5
C5—C6—C1	119.5 (6)	O6—C17—O5	125.5 (6)
C5—C6—C7	118.3 (6)	O6—C17—N4	125.0 (6)
C1—C6—C7	122.2 (6)	O5—C17—N4	109.5 (5)
N1—C7—C6	119.5 (6)	O5—C18—H18A	109.5
N1—C7—H7	120.2	O5—C18—H18B	109.5
C6—C7—H7	120.2	H18A—C18—H18B	109.5
O2—C8—O3	125.8 (6)	O5—C18—H18C	109.5
O2—C8—N2	123.9 (6)	H18A—C18—H18C	109.5
O3—C8—N2	110.4 (5)	H18B—C18—H18C	109.5
O3—C9—H9A	109.5	C7—N1—N2	118.2 (5)
O3—C9—H9B	109.5	C8—N2—N1	118.3 (5)
H9A—C9—H9B	109.5	C8—N2—H2	120.9
O3—C9—H9C	109.5	N1—N2—H2	120.9
H9A—C9—H9C	109.5	C16—N3—N4	118.5 (5)
H9B—C9—H9C	109.5	C17—N4—N3	117.7 (5)
O4—C10—C11	119.4 (6)	C17—N4—H4	121.2
O4—C10—C15	122.1 (6)	N3—N4—H4	121.2
C11—C10—C15	118.5 (6)	C1—O1—H1	109.5
C12—C11—C10	121.8 (6)	C8—O3—C9	116.1 (5)
C12—C11—Br3	119.5 (6)	C10—O4—H4A	109.5
C10—C11—Br3	118.6 (5)	C17—O5—C18	116.5 (5)
			
O1—C1—C2—C3	179.0 (6)	C11—C12—C13—C14	−0.3 (11)
C6—C1—C2—C3	−0.1 (10)	C11—C12—C13—Br4	177.0 (5)
O1—C1—C2—Br1	1.2 (8)	C12—C13—C14—C15	0.5 (10)
C6—C1—C2—Br1	−177.9 (4)	Br4—C13—C14—C15	−176.8 (5)
C1—C2—C3—C4	0.6 (11)	C13—C14—C15—C10	0.0 (10)
Br1—C2—C3—C4	178.5 (5)	C13—C14—C15—C16	178.2 (6)
C2—C3—C4—C5	0.1 (11)	O4—C10—C15—C14	179.8 (5)
C2—C3—C4—Br2	−179.3 (5)	C11—C10—C15—C14	−0.6 (9)
C3—C4—C5—C6	−1.3 (10)	O4—C10—C15—C16	1.7 (9)
Br2—C4—C5—C6	178.1 (5)	C11—C10—C15—C16	−178.7 (6)
C4—C5—C6—C1	1.8 (9)	C14—C15—C16—N3	−173.7 (6)
C4—C5—C6—C7	−177.7 (6)	C10—C15—C16—N3	4.4 (9)
O1—C1—C6—C5	179.8 (6)	C6—C7—N1—N2	−177.8 (5)
C2—C1—C6—C5	−1.1 (9)	O2—C8—N2—N1	1.6 (10)
O1—C1—C6—C7	−0.7 (9)	O3—C8—N2—N1	−178.3 (5)
C2—C1—C6—C7	178.4 (6)	C7—N1—N2—C8	176.3 (6)
C5—C6—C7—N1	173.9 (6)	C15—C16—N3—N4	177.9 (5)
C1—C6—C7—N1	−5.7 (9)	O6—C17—N4—N3	−2.1 (10)
O4—C10—C11—C12	−179.6 (6)	O5—C17—N4—N3	178.5 (5)
C15—C10—C11—C12	0.8 (10)	C16—N3—N4—C17	−176.3 (6)
O4—C10—C11—Br3	−0.7 (8)	O2—C8—O3—C9	1.5 (10)
C15—C10—C11—Br3	179.7 (4)	N2—C8—O3—C9	−178.6 (6)
C10—C11—C12—C13	−0.4 (10)	O6—C17—O5—C18	−2.5 (10)
Br3—C11—C12—C13	−179.3 (5)	N4—C17—O5—C18	176.9 (5)

### Hydrogen-bond geometry (Å, °)

**Table:**

Cg1 and Cg2 are centroids of the C1–C6 and C10–C15 rings, respectively.

**Table:**

*D*—H···*A*	*D*—H	H···*A*	*D*···*A*	*D*—H···*A*
O1—H1···N1	0.82	1.88	2.604 (7)	146
N2—H2···O6	0.86	2.01	2.845 (6)	163
N4—H4···O2^i^	0.86	2.00	2.828 (6)	161
O4—H4A···N3	0.82	1.89	2.609 (7)	145
C7—H7···O6	0.93	2.56	3.312 (8)	139
C16—H16···O2^i^	0.93	2.52	3.282 (8)	139
C9—H9B···Cg1^ii^	0.97	2.89	3.594 (8)	131
C18—H18C···Cg2^iii^	0.97	2.89	3.561 (8)	128

Symmetry codes: (i) *x*, *y*−1, *z*; (ii) −*x*, −*y*+2, −*z*+1; (iii) −*x*+1, −*y*+1, −*z*+1.
